# The cerebellar phenotype of Charcot-Marie-Tooth neuropathy type 4C

**DOI:** 10.1186/s40673-019-0103-8

**Published:** 2019-07-15

**Authors:** Humberto Skott, Cristina Muntean-Firanescu, Kristin Samuelsson, Luca Verrecchia, Per Svenningsson, Helena Malmgren, Carmen Cananau, Alberto J. Espay, Rayomand Press, Göran Solders, Martin Paucar

**Affiliations:** 1 Department of Neurology, Karolinska University Hospital, Stockholm, Sweden; 2 Department of Neurophysiology, Karolinska University Hospital, Stockholm, Sweden; 3 Department of Clinical Neuroscience, Karolinska Institutet Stockholm, Stockholm, Sweden; 4 Trauma and Reparative Medicine Theme, Karolinska University Hospital, Stockholm, Sweden; 5 ENT unit, Department of Clinical Science, Intervention and Technology (CLINTEC), Karolinska Institutet Stockholm, Stockholm, Sweden; 6 Department of Genetics, Karolinska University Hospital, Stockholm, Sweden; 7 Department of Molecular Medicine and Surgery, Karolinska Institutet, Stockholm, Sweden; 8 Department of Nuclear Medicine, Karolinska University Hospital, Stockholm, Sweden; 9 Department of Neurology, Gardner Neuroscience Institute, Gardner Center for Parkinson’s Disease and Movement Disorders, University of Cincinnati, Cincinnati, OH USA

**Keywords:** Charcot-Marie-tooth neuropathy type 4C, *SH3TC2*, Ataxia, Friedreich-like ataxia, vHIT, VEMP

## Abstract

**Background:**

Friedreich ataxia (FRDA) is the most common familial ataxia syndrome in Central and Southern Europe but rare in Scandinavia. Biallelic mutations in SH3 domain and tetratricopeptide repeats 2 (*SH3TC2)* cause Charcot-Marie-Tooth disease type 4C (CMT4C), one of the most common autosomal recessive polyneuropathies associated with early onset, slow disease progression and scoliosis. Beyond nystagmus reported in some patients, neither ataxia nor cerebellar atrophy has been documented as part of the CMT4C phenotype.

**Methods:**

Here we describe a single centre CMT4C cohort. All patients underwent a comprehensive characterization that included physical examination, neurophysiological studies, neuroimaging and genetic testing. In a patient with cerebellar features, an evaluation of the vestibular system was performed.

**Results:**

All five patients in this cohort harbored the R954X mutation in *SH3TC2* suggesting a founder effect*.* Two patients had been diagnosed as FRDA. One of them, an 80-year-old woman had onset of unsteadiness during childhood leading to gradual loss of mobility. She also had scoliosis and hearing loss. On examination she had generalized muscle atrophy, leg flaccidity, pes cavus, facial myokymia, limb dysmetria, dysarthria and gaze-evoked nystagmus. She exhibited bilateral vestibular areflexia. Neuroimaging demonstrated atrophy in the frontoparietal regions and cerebellar hemispheres.

**Conclusions:**

CMTC4A may present with a cerebellar phenotype and mimic a flaccid-ataxic form of FRDA. Absence of cardiomyopathy or endocrine abnormalities and lack of pathological dentate iron accumulation in CMT4C distinguish it from FRDA.

**Electronic supplementary material:**

The online version of this article (10.1186/s40673-019-0103-8) contains supplementary material, which is available to authorized users.

## Introduction

Friedreich ataxia (FRDA) is the most common autosomal recessive ataxia in Western Europe, the Middle East and Northern Africa (1 in 40,000) whereas Charcot-Marie-Tooth type 4C (CMT4C) is one of the most prevalent (18%) type of autosomal recessive CMT [1]. CMT4C is caused by biallelic mutations in the SH3 domain and tetratricopeptide repeats 2 (*SH3TC2)* and has been associated with a heterogeneous clinical presentation [1–3]. Some clinical features of FRDA and CMT4C overlap, with both conditions featuring polyneuropathy and scoliosis. We reviewed a local cohort of 5 patients with CMT4C and suggest that (a) cerebellar signs associated with cerebellar atrophy may be considered within the phenotypic spectrum of CMT4C and (b) its early stages can mimic a “flaccid-ataxic” form of FRDA with slower progression, milder cerebellar ataxia and prominent leg flaccidity (due to severe demyelinating polyneuropathy rather than sensory ganglionopathy). In addition, our cases include trigeminal nerve enlargement and frontoparietal atrophy which are novel neuroimaging abnormalities in CMT4C.

## Patients and methods

This study was approved by the local ethics committee in Stockholm; patients provided oral and written consent. Five unrelated CMT4C patients were evaluated using standard motor scales and peripheral nerve conduction studies. Validated scales used included the functional ataxia staging score of overall mobility (subscore of Friedreich’s Ataxia Rating Scale-FARS), the Scale for the Assessment and Rating of Ataxia (SARA) and a composite subscale of the CMT Neuropathy Score (version 2) based on symptoms and signs (CMTES) [4–6]. All patients underwent also neuroimaging and targeted gene panel analyses; one patient was investigated with mass sequencing (patient 5 in Table 1). Briefly, genomic DNA from patient 5 was subjected to massive parallel sequencing with whole genome sequencing. The sequencing and primary filtering of variants were performed at Clinical genomics, SciLife, Solna, Sweden. Sequence data was mapped to reference sequence [GRCh37/UCSChg19], and 99% achieved at least 20x coverage. Identified sequence variants were analyzed and filtered using designated software (SCOUT, Clinical Genomics, SciLife Solna). Known genes associated with ataxia and neuromuscular disorders were analyzed. Sequences in exons and exon/intron boundaries, and variants with population frequencies < 0.01 were interpreted.

**Table 1:** Clinical and neurophysiological features

Patient	1	2	3	4	5
Sex/Current age (years)	M/50	F/73	F/58	M/32	F/81
Ethnicity	Swedish	Swedish	Swedish	Central European^a^/Swedish	Swedish
Age of onset/Disease duration (years)	7/43	6/67	7/51	6/26	9/72
Initial symptoms	Feet supination	Unsteady gait	Unsteady gait	Running difficulties	Toe walking
LL paresis distal/ proximal	Severe/Moderate	Severe/Normal	Moderate/Absent	Mild/Absent	Severe/Moderate
UL paresis distal/proximal	Severe/Moderate	Moderate/Normal	Mild/Absent	Absent	Moderate/Absent
Walking disability	Wheelchair	Sticks	Minimal disability	Minimal disability	Wheelchair
Deep tendon reflexes	Absent	Absent	Absent	Absent	Absent
Pinprick sensation	Absent to the knee + reduced to the wrists	Reduced to the ankles + wrists	Reduced to the ankles +wrists	Reduced to the ankles + wrists	Reduced to the ankles + wrists
Vibration sense	Absent at knee	Absent at knee	Absent at knee	Absent at knee	Reduced to sternum
Pes cavus	Y	Y	Y	Y	Y
Scoliosis	Y	Y	Y	Y	Y
Hearing loss	Y	Y	Y	Y	Y
Eye movement abnormalities	Horizontal gaze evoked nystagmus, hypermetric saccades	Horizontal gaze evoked nystagmus,	Absent	Square wave jerks on fixation, horizontal gaze evoked nystagmus,	Hypometric saccades, gaze-evoked nystagmus,
Other features	Dysphagia, aspiration pneumonia in later stages	Trigeminal neuralgia	Tremor, trigeminal neuralgia	Urinary incontinence	Dysarthria Dysphagia
FARS	5	4	1	1	5
SARA (age of last exam, years)	22 (50)	8.5 (72)	4.5 (57)	5 (30)	30.5 (78)
CMTES	28	13	9	10	19
NCS	Demyelinating neuropathy	Demyelinating neuropathy	Demyelinating neuropathy	Demyelinating neuropathy	Axonal and demyelinating neuropathy
Brain MRI	Normal	Normal	Bilateral thickening of trigeminal nerves	Normal	Cerebellar and frontoparietal atrophy

The course of disease was slow in most cases, however in patient 1 it has been faster and more aggressive making him wheel-chair bound at age 50. All patients harbor the homozygous R954X mutation in *SH3TC2*. ^a^ One parent was Hungarian. Key: *M* Male, *F* Female, *n/a* Not available, *LL* Lower limbs, *UL* Upper limbs, *Y* Yes, *N No, FARS* NA, no assessed, Friedreich’s Ataxia Rating Scale, *SARA* Scale for the Assessment and Rating of Ataxia, *CMTES* Charcot-Marie-Tooth examination score, *NCS* Nerve conduction studies

### Vestibular assessment

The vestibular function was documented on a patient with cerebellar features (patient 5) with bithermal caloric test according to Fitzgerald-Hallpike, video head impulse test (vHIT) and with cervical vestibular evoked myogenic potentials (cVEMP) evoked by air conducted stimuli [7]. Through this test battery we investigated the vestibular ocular reflex pathway (VOR) using caloric test and vHIT and the vestibular cervical reflex pathway (VCR), using cVEMP and vHIT.

## Results

Mass sequencing in patient 5 revealed a homozygous R954X mutation in the *SH3TC2* gene. The same homozygous mutation was found in 4 other patients (aged 32–80 years) confirming the diagnosis of CMT4C. Disease onset was during childhood in all cases (aged 6–9 years). Two patients in this cohort had been misdiagnosed as FRDA during childhood (Patients 2 and 5). One of them had cerebellar atrophy (reported below). Another had neuralgia symptoms and MRI evidence of thickening of both trigeminal nerves (Table 1). The course of disease was slow in most cases; however patient 1 (age 50) had an aggressive course making him wheel-chair bound much earlier than the other patients in this cohort.

### Case description

**Fig. 1:**
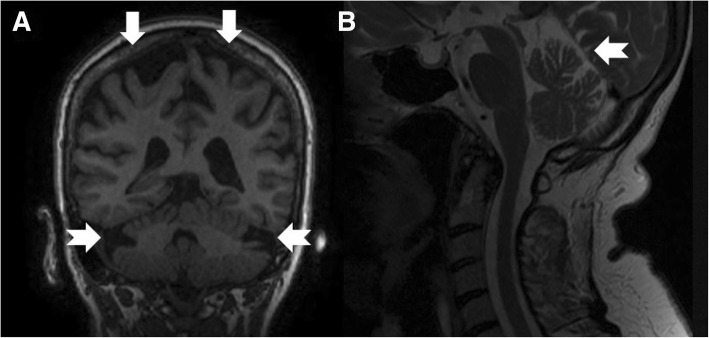
Coronal T1-weighted (**a**), mid-sagittal T2-weighted (**b**) of patient 5 at age 76. Note atrophy in the bi-parietal brain regions (thick arrows) as well as lateral hemispheres and superior vermis of the cerebellum (notched arrows)

**Fig. 2:**
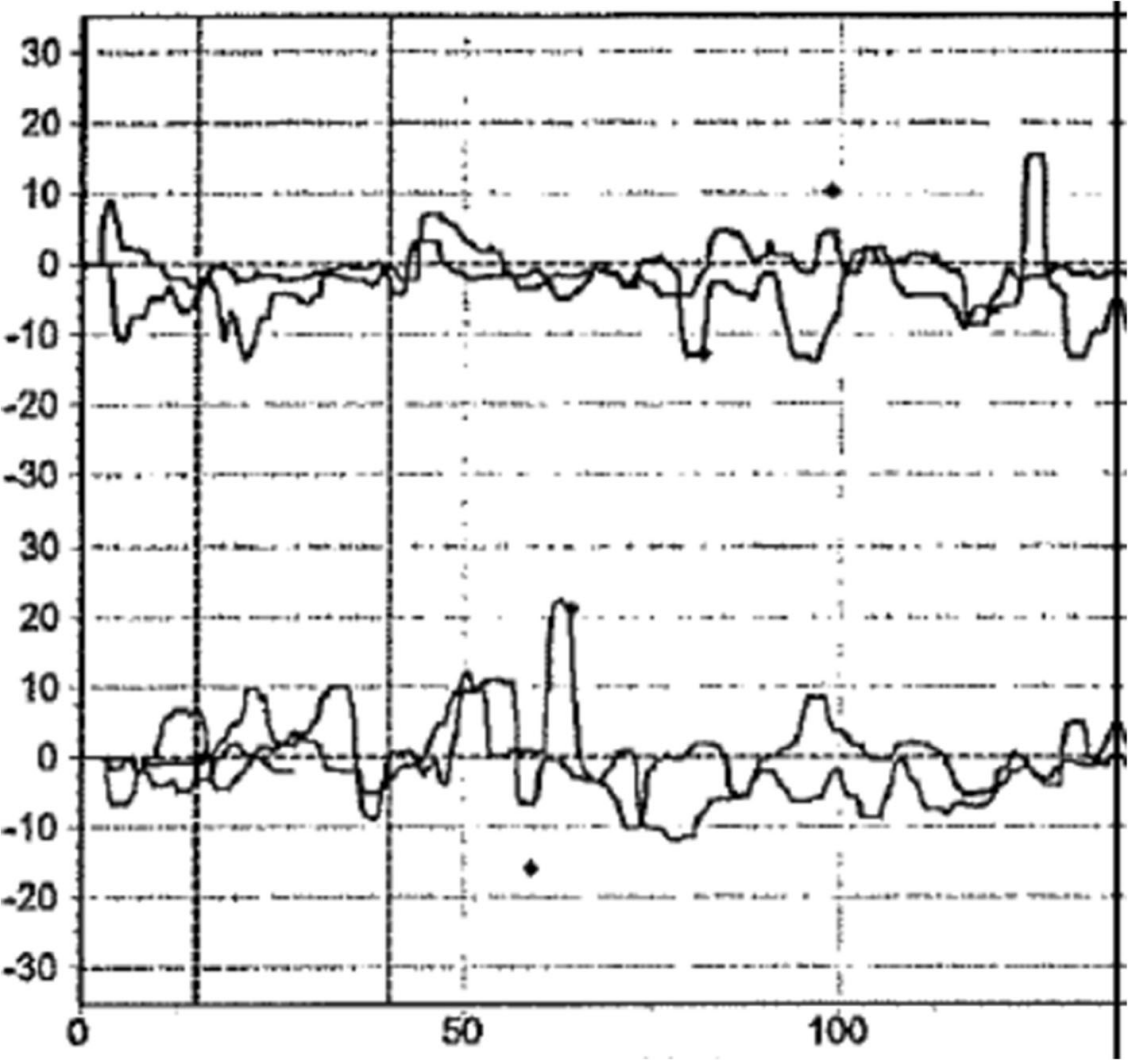
Calorigram for the right irrigations (on the top) and left irrigations (on the bottom). The two overlapping traces for each side correspond to the videooculography recording after cold and warm irrigation, in terms of instantaneous eye angular velocity: ^0^ (Y-axis) over time in seconds (X-axis). The two traces for each side configure an irregular eye motility in darkness in the absence of a consistent caloric nystagmus pattern. This pattern is typical for bilateral vestibular failure

**Fig. 3:**
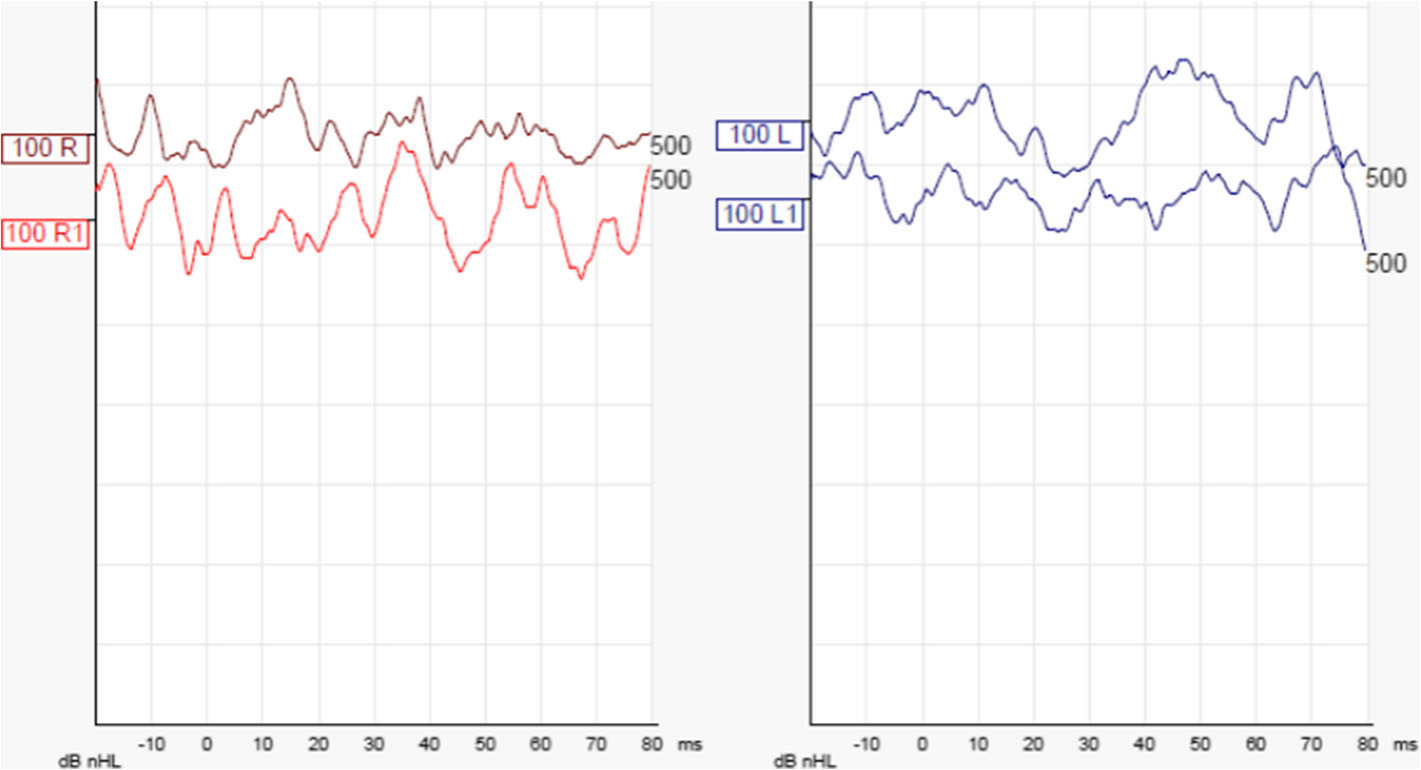
cVEMP evoked by submaximal air conducted 500 Hz tone bursts, delivered at the right ear (left panel) and left ear (right panel). Responses recorded at the ipsilateral sternocleidomastoideus muscle under controlled contraction. The two traces per side represent the averaged response of 120 sweeps. The classical positive-negative EMG deflection within 12–25 ms after stimuli is not identifiable on either a side with responses configuring EMG noise only

**Fig. 4:**
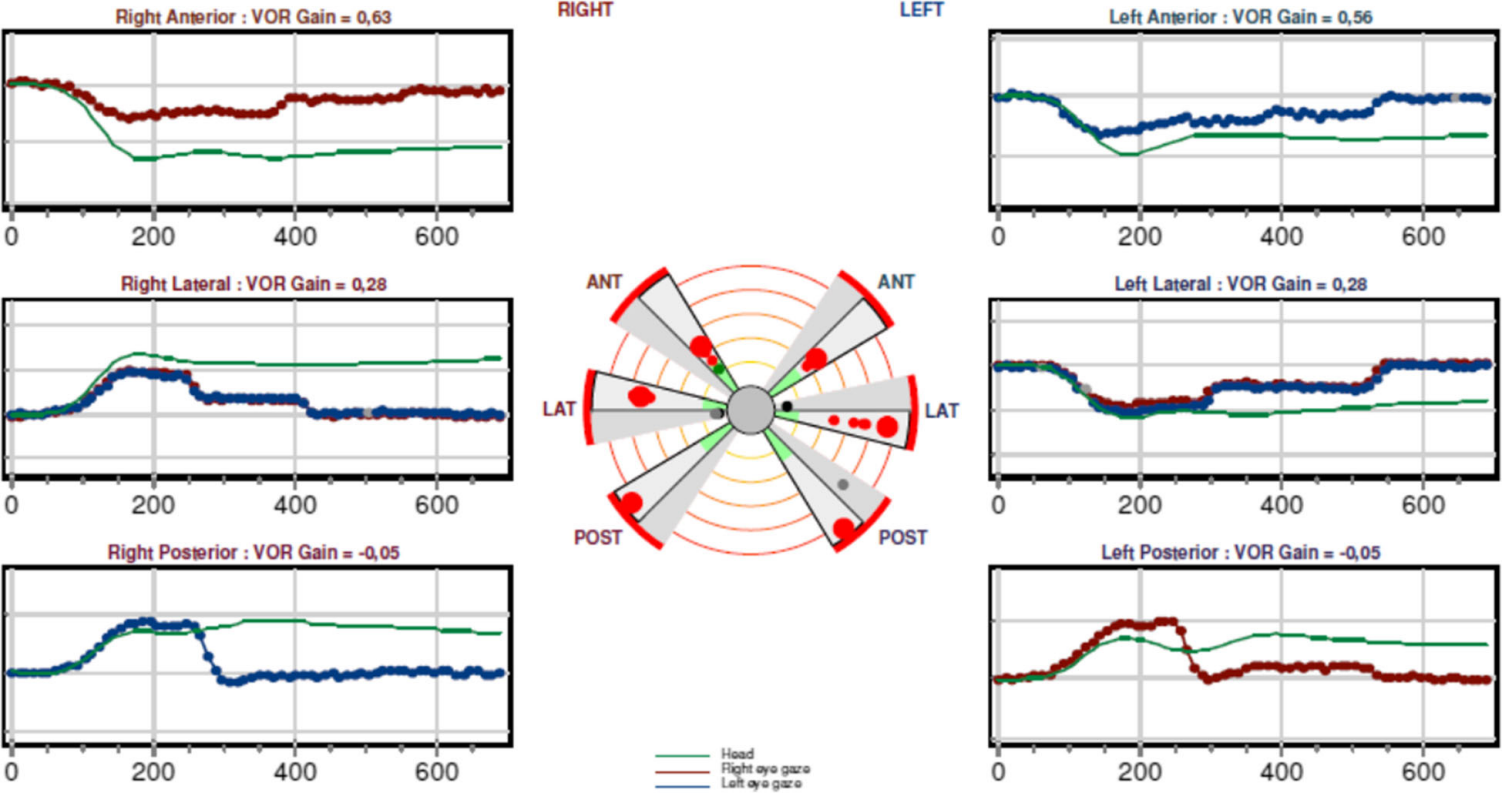
Summary of findings on vHIT. Test conducted with the system Synapsis vHIT Ulmer 2. The canalogram at the center shows a general VOR gain depression for all the tested canals bilaterally. Selected responses (corresponding to the larger spots on the canalogram) are shown in panels, with the eye position (dotted lines) traced in contrast with the head position (green lines) over time (ms). VOR gains are given for the specific trials on the top of the panels


This 80-year-old Swedish woman (Patient 5), born to non-consanguineous parents, had early-onset of unsteadiness and gait difficulties during childhood leading to gradual loss of mobility. She needed a cane at age 50, followed by walker, and became wheelchair-bound by age 60. She developed scoliosis, restrictive pulmonary dysfunction, macular degeneration and sensorineural hearing loss, requiring hearing aids. Her symptoms included numbness, paresthesia with pain and discomfort in both legs, and tongue burning, relieved with gabapentin. Despite these, she had worked part-time in an office. The patient also reported slurred speech and mild dysphagia. On exam, she exhibited leg amyotrophy, foot drop, upper limb and facial myokymia, gaze-evoked and hypermetric saccades mild appendicular dysmetria and dysarthria (Additional file 1: Video). Previously, side-changing nystagmus was documented in her medical records. There was no evidence of spasticity or other pyramidal signs, the limbs were rather flaccid especially the legs. The Montreal Cognitive Assessment score was 26/30. Brain MRI showed atrophy in frontal and parietal brain regions and in the cerebellar hemispheres (Fig. 1a and b). Electroneurography and quantitative sensory testing demonstrated a severe axonal and demyelinating sensorimotor polyneuropathy involving thin sensory fibers. Electromyograpy demonstrated reduction of motor units and myokymia with spontaneous regular rhythmic discharges of motor units in triplets or quadruples in facial muscles. In addition, myokymia activity was recorded in the facial muscles. The vestibular assessment demonstrated a very poor vestibular function compatible with bilateral vestibular areflexia: absence of caloric nystagmus (Fig. 2) or elicitation of dizziness/vertigo upon bithermal caloric irrigation. In addition, the vHIT revealed a very poor gain for the stimulation of the posterior and lateral canals and a depressed but still consistent, response with stimulation of the anterior semicircular canals in both sides. The VOR gain measured with vHIT had an average of 0.29 (Fig. 3). The cVEMP did not demonstrate reproducible responses at two different trails each side (Fig. 4). After excluding FRDA, polyglutamine-related spinocerebellar ataxias, and duplication/deletion in the *PMP22* gene, we proceeded with whole exome sequencing, which detected the pathogenic homozygous mutation c.2860C > T (pArg954*, R954X) in *SH3TC2*. This is the most common CMT4C mutation reported to date [1, 2, 8].

**Additional file 1:** Video S1. The patient is shown during an evaluation at the age 78 years. She is confined to a wheelchair with inability to stand due to flaccid areflexia in the legs associated with foot drop. The video segment demonstrates mild dysarthria, myokymia in the distal upper limbs and facial muscles. There is intermittent postural hand tremor. Other features shown are mild dysmetria, saccadic pursuit, bilateral gaze-evoked nystagmus, and hypermetric saccades. (MP4 250759 kb)


## Conclusions

The long disease duration, type of polyneuropathy leading to flaccid ataxia, and absence of systemic features such as cardiomyopathy, diabetes and optic atrophy suggested a diagnostic revision from FRDA in two of five CMT4C patients. While cerebellar atrophy occurs at later stages, an important imaging feature in FRDA is the presence of atrophy and iron accumulation in the dentate nuclei [9]. This is the first report of cerebellar atrophy in CMT4C; however this finding has to be confirmed in larger cohorts.

When polyneuropathy is a predominant initial feature in FRDA, leading to flaccidity, some patients may be misdiagnosed as CMT [10, 11]. However, neurophysiology is helpful to distinguish the sensory ganglionopathy of FRDA from the demyelinating neuropathy of CMT4C. Spasticity is variable in FRDA, becoming more prominent in later stages of the disease (affecting 20% of cases); flaccidity is more common in the atypical forms of the disease, affecting 30–40% of cases [11]. Besides sensory ataxia, other overlapping features for CMT4C and FRDA include scoliosis and hearing loss (Table 2).

**Table 2:** Comparison between Friedreich ataxia and Charcot-Marie-Tooth neuropathy type 4C

Features	Friedreich ataxia (FRDA)	Charcot-Marie-Tooth neuropathy type 4C (CMT4C)
Age of onset	Childhood-adolescence	Childhood
Course of disease	Typically early loss of mobility	Loss of mobility in some
Prognosis	Reduced life span	Life expectancy unaffected
Scoliosis	Y (Very common)	Y
Type of polyneuropathy	Axonal sensory	Demyelinating motor and sensory
Myokymia	N	Y
Foot deformity	Y	Y
Hearing loss	Y (20%)	Y (12%) [3]
Eye movement abnormalities	Square wave jerks most commonly [12]	Nystagmus in a few patients
Vestibular signs	Y	Y
Risk for diabetes	Y (20%)	N
Hypertrophic cardiomyopathy	Y [13]	N
Respiratory failure	N	Y
Cerebellar atrophy	In late stages	Very rare
Spinal cord atrophy	Y	Unknown
Other radiological abnormalities	Iron accumulation in the dentate nuclei Atrophy of dentate nuclei [9]	Thickening of cranial nerves
Pathology of the brain, spinal cord and dorsal roots	Depletion of myelinated fibers in posterior columns, neuronal loss in the dorsal nuclei of Clarke columns thinning of dorsal roots and spinal cord [14], progressive atrophy of the dentate nucleus [15]	Unknown

Our experience with CMT4C suggests that this entity includes an areflexic, hypotonic FRDA-like phenotype. It is important to note that ataxia has also been reported in other CMT variants, such as X-linked CMTX5 [16] although cerebellar atrophy is otherwise rare in the CMT spectrum. While early features of CMT4C can mimic a flaccid form of FRDA, CMT4C exhibits slower progression, milder cerebellar ataxia, and more severe flaccidity in the legs as a result of severe demyelinating polyneuropathy (Table 2). Another important difference between CMT4C and FRDA are determined by the pattern of abnormal eye movements. Square wave jerks are the most common oculomotor abnormality in FRDA [10–12] whereas the presence of nystagmus in CMT4C is rare. Ocular flutter has been reported once in CMT4C [1]. Only a previous study demonstrated subtle vestibulopathy in some patients with CMT4C; however none of those patients had gaze-evoked nystagmus or other features suggesting cerebellar dysfunction [17]. Even though we found evidence of causal or contributory vestibulopathy to explain nystagmus in patient 5, she had other clear cerebellar abnormalities (dysarthria, dysphagia and cerebellar atrophy). Dysmetria in this case is reasonably a manifestation of sensory and cerebellar dysfunction. According to our results on patient 5, a vestibular areflexia is an element of this syndrome. This type of vestibular dysfunction was reported in 7 of 10 Spanish patients with CMT4C [17]. Even though a general depression of vestibular responses occurs with advanced age [18, 19], the very poor vestibular response demonstrated with different modalities is consistent with an underlying bilateral vestibular failure in this patient. Interestingly, a consistent difference in VOR defect for the vertical impulses is observed in our patient, with much more depressed VOR gain in the upward impulses compared with the downward impulses. This vHIT pattern could be the expression of oculomotor disorders associated to a general vestibular failure [20]. Thus, the global vestibular depression is likely due to central dysregulation of oculomotor pathways combined with vestibular insufficiency. Cerebellar ataxia with neuropathy and vestibular areflexia syndrome (CANVAS) is another consideration as a differential diagnosis. However, neither onset during childhood nor hearing loss is part of the CANVAS phenotype [21]. Taken together our findings are of clinical relevance when considering the low prevalence of FRDA in Scandinavia compared to other parts of Europe [22].

In line with previous findings our data suggest a founder effect for our cohort [4]. An additional novelty in the spectrum of mutations in *SH3TC2* is thickening of trigeminal nerves in one CMT4C patient with facial pain. Facial pain has been described before in CMT4C [1]; furthermore widespread cranial nerve enlargement has been reported otherwise in CMT1A [23]. Expression of *SH3TC2* is ubiquitous and includes the cerebellum, but pathological studies to date have focused on peripheral nerves only. Neuropathological brain studies will be needed in order to further define the clinico-anatomical correlations of ataxia and vestibular dysfunction in CMT4C patients.

## Data Availability

Not applicable.

## Abbreviations

CMT4C: Charcot-Marie-Tooth disease type 4C
cVEMP: Cervical vestibular evoked myogenic potentials
*SH3TC2*: SH3 domain and tetratricopeptide repeats 2
vHIT: Video head impulse test
